# GPT-4o–Powered Preanesthetic AI: Development and Validation Study

**DOI:** 10.2196/83893

**Published:** 2026-08-25

**Authors:** Bo-Han Lin, Cheng-Wei Lu, I-Ching Hou, Tzu-Yu Lin, Chin-E Liu, Chong-Sun Khoi

**Affiliations:** 1 Department of Anesthesiology, Far Eastern Memorial Hospital, No. 21, Section 2, Nanya S. Road, Banqiao District, New Taipei City, 220, Taiwan, +886-2-8966-7000 ext 4371; 2 Department of Nursing, National Yang Ming Chiao Tung University, Taipei City, Taiwan; 3 Department of Mechanical Engineering, College of Engineering, Yuan Ze University, Taoyuan City, Taiwan; 4 Graduate School of Biotechnology and Bioengineering, College of Medicine and Nursing, Yuan Ze University, Taoyuan City, Taiwan

**Keywords:** preanesthetic assessment, AI, GPT-4o, American Society of Anesthesiologists, ASA classification, postoperative nausea and vomiting, PONV, electronic medical records, large language models

## Abstract

**Background:**

Accurate preanesthetic assessment is essential for perioperative risk stratification, but conventional tools such as the American Society of Anesthesiologists (ASA) physical status classification and postoperative nausea and vomiting (PONV) risk scores may be affected by subjective judgment, incomplete documentation, and fragmented clinical data. Large language models may support preanesthetic assessment by integrating structured and unstructured clinical information.

**Objective:**

This study aimed to develop and retrospectively validate a GPT-4o (OpenAI)–powered AI system for preanesthetic assessment. The primary validation focus was the agreement between AI-generated and clinician-assigned ASA physical status classifications. PONV risk stratification was evaluated as an additional clinically relevant performance outcome. A secondary objective was to assess the incremental contribution of National Health Insurance (NHI) cloud data to model performance.

**Methods:**

This single-center retrospective validation study included 600 adult surgical patients randomly sampled from 4404 eligible inpatient surgical patients between January and May 2025. The system processed structured and unstructured electronic health record data, with and without NHI cloud data, to generate preanesthetic assessment outputs. ASA agreement was assessed using Cohen κ with 95% CIs. PONV prediction was evaluated using sensitivity, specificity, overall accuracy, positive predictive value, negative predictive value, confusion matrix analysis, the 2-sided Fisher-Freeman-Halton exact test, and Cramér V.

**Results:**

Among the 600 patients, clinician-assigned ASA classifications were ASA I in 39 patients, ASA II in 498 patients, ASA III in 57 patients, and ASA IV in 6 patients. Agreement between AI-generated and clinician-assigned ASA classifications was high when NHI data were incorporated (κ=0.883, 95% CI 0.841‐0.946), whereas agreement was lower without NHI data (κ=0.518, 95% CI 0.412‐0.691). A total of 49 (8.2%) patients experienced documented PONV within 24 hours after surgery. Using the high-risk category as the primary test-positive threshold, the model achieved a sensitivity of 34.7% (95% CI 22.9‐48.7), a specificity of 99.1% (95% CI 97.9‐99.6), a positive predictive value of 77.3%, and a negative predictive value of 94.5%. The association between the model-assigned PONV risk categories and observed PONV outcomes was statistically significant (*P*<.001, Cramér V=0.531).

**Conclusions:**

The GPT-4o–powered preanesthetic assessment system demonstrated feasibility and high agreement with clinician-assigned ASA classifications when NHI cloud data were incorporated. For PONV risk stratification, the system showed high specificity and a high negative predictive value but modest sensitivity, indicating that it identified a small high-risk subgroup with a high observed PONV incidence but did not capture all patients who developed PONV. These findings support the use of large language model–based decision-support tools as adjuncts to, rather than replacements for, anesthesiologist judgment.

## Introduction

Accurate preanesthetic assessment is fundamental to perioperative safety because it supports early identification of patients at increased risk for anesthesia-related complications and informs appropriate perioperative planning. In routine practice, the American Society of Anesthesiologists (ASA) physical status classification system and the Apfel risk score for postoperative nausea and vomiting (PONV) are among the most commonly used tools for preoperative risk stratification. Despite their widespread use, both instruments have important limitations that may affect their consistency and scalability in contemporary clinical workflows.

The ASA classification system remains clinically useful but is inherently dependent on clinician judgment. Prior studies have shown substantial variability in ASA assignment across providers, even when similar clinical information is available [1,2]. This variability is not limited to anesthesiologists. In procedure-based specialties, surgeons may assign lower ASA classes than AI-assisted or anesthesia-based assessments, suggesting that risk may be underestimated when classification is performed outside a dedicated anesthetic context [3]. Likewise, the Apfel score is practical and well established, but its predictive performance depends on the availability of a small set of clinical risk factors, including nonsmoking status, prior PONV, and motion sickness history, which may be incompletely documented in routine electronic records [4]. Together, these limitations illustrate a broader challenge in perioperative medicine: clinically important preanesthetic judgments are often made using fragmented data, subjective interpretation, or both.

AI offers a potential solution to this problem by enabling more standardized and data-driven preoperative assessment. In recent years, machine learning and natural language processing approaches have demonstrated promising performance in perioperative risk modeling, including postoperative mortality prediction and automated ASA-related classification using preoperative electronic health record (EHR) data [1,5]. Beyond anesthesia-specific applications, AI-based models have also shown feasibility for preoperative risk stratification in other clinical domains, such as computed tomography–based risk classification of gastrointestinal stromal tumors [6]. More recently, large language models (LLMs) have emerged as particularly attractive tools because they can process not only structured variables but also unstructured clinical narratives, which are abundant in real-world perioperative documentation. This capacity is especially relevant in anesthesiology, where clinically meaningful information is frequently embedded in free-text records such as admission notes, consultation summaries, medication histories, and operative documentation.

Among current LLMs, GPT-4o (OpenAI) has shown strong performance across medical reasoning tasks. Published evaluations have reported variable GPT-4o performance across medical reasoning tasks, including radiology diagnostic quiz cases and high-stakes anesthesiology examination settings [7-9]. These findings suggest that model performance varies with task structure and domain specificity and may be sufficient to support clinically relevant reasoning under controlled conditions. At the same time, these studies also highlight an important caution: high overall performance does not eliminate the possibility of clinically meaningful error. In anesthesiology-focused benchmarking, unsupported medical claims were a frequent error type among incorrect responses, underscoring the need for careful validation before clinical deployment [8].

Several strategies have been proposed to improve the reliability of LLM-based medical systems. Retrieval-augmented generation, for example, can anchor model outputs to external knowledge sources and has been shown to improve accuracy in preoperative fitness assessments [10]. In parallel, conventional machine learning models for PONV prediction have outperformed traditional clinical scores in some datasets [11]. However, many existing predictive systems remain limited by their dependence on structured inputs and may not fully exploit the rich narrative content available in EHRs. LLMs may offer an advantage in this respect, but their real-world clinical validity in perianesthetic workflows remains insufficiently studied.

Another unresolved issue concerns how clinicians interact with AI-generated recommendations. Human-AI collaboration can improve diagnostic performance when AI is used to augment rather than replace expert judgment [12]. However, clinician acceptance of AI outputs should not be interpreted as evidence of correctness. Recent work has shown that physician satisfaction with LLM responses does not necessarily correlate with response accuracy, raising concerns that automation bias may obscure clinically important errors if human oversight is weak [13]. For this reason, evaluating LLMs in real clinical environments requires not only accuracy testing but also careful attention to workflow integration and the preservation of clinician accountability.

The objective of this study was to develop and retrospectively validate a GPT-4o–powered AI-assisted preanesthetic assessment system. The primary validation focus was the agreement between AI-generated and clinician-assigned ASA physical status classifications. PONV risk stratification was evaluated as an additional clinically relevant performance outcome. A secondary objective was to quantify the incremental effect of incorporating National Health Insurance (NHI) cloud data on model performance. This study is reported in accordance with the TRIPOD (Transparent Reporting of a Multivariable Prediction Model for Individual Prognosis or Diagnosis)-LLM guidance for studies using LLMs, with additional consideration of the broader TRIPOD+AI reporting framework for prediction model studies [14,15].

## Methods

### Study Design and Setting

This was a single-center retrospective validation study conducted at Far Eastern Memorial Hospital. The study evaluated a GPT-4o–enabled preanesthetic assessment system using existing clinical records from surgical patients who underwent preanesthetic consultation during the study period. The system integrated structured and unstructured data from hospital information systems and NHI cloud records to generate preanesthetic assessment outputs, including ASA physical status classification and PONV risk stratification.

### Ethical Considerations

This study was reviewed and approved by the Institutional Review Board of Far Eastern Memorial Hospital (IRB 114151-E). Because this was a retrospective study using existing clinical records, the requirement for additional study-specific informed consent was waived by the Institutional Review Board. At admission, all patients provided written consent for the institutional use of their personal and medical data for purposes including clinical care, research, automated data processing, and related quality improvement activities, in accordance with institutional policy.

To protect privacy and confidentiality, direct identifiers, including patient names, were removed or replaced with generic placeholders before transmission to the cloud-based AI service. The field labeled “date of birth” contained only the patient’s year of birth; the month and day were not transmitted. Limited demographic variables, including age, sex, blood type, and year of birth, were retained in the transmitted data. AI-generated outputs were mapped back to identifiable clinical records only within the hospital’s secure local database.

No compensation was provided to participants because the study was retrospective, involved no direct participant contact, and used existing clinical data. Figures and supplementary materials were prepared using system architecture diagrams and workflow diagrams. No identifiable individual participant images or personal information is included in the manuscript or supplementary materials.

### Data Privacy and Security

To protect patient privacy and support regulatory compliance, a multilayered security framework was implemented. First, direct patient identifiers were removed or replaced with generic placeholders on-premises before transmission to the cloud-based AI service. Patient names were replaced with generic placeholders, and the birth-date field contained only the year of birth; the month and day were excluded. Limited demographic variables required for clinical assessment, including age, sex, blood type, and year of birth, were retained. Second, transmitted data were protected using encryption mechanisms during data transfer. Third, the hospital maintained a formal contractual agreement, with the AI service provider prohibiting the use of institutional data for external model training or third-party purposes. Fourth, AI-generated results were mapped back to identifiable clinical records exclusively within the hospital’s secure local database after retrieval.

### System Architecture and Workflow

**Figure 1.**
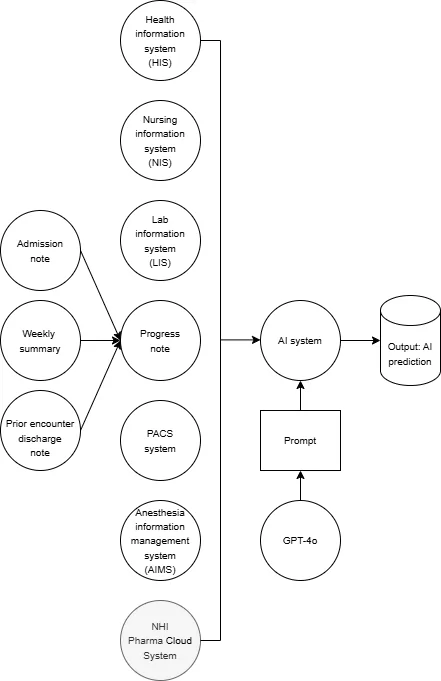
The system architecture supports GPT-4o–enabled preanesthetic assessment. The model integrates structured and unstructured preoperative data from the hospital information system, nursing information system, laboratory information system, and anesthesia information management system, as well as National Health Insurance (NHI) cloud data. Unstructured inputs included preoperative clinical documentation and historical summaries from prior hospital encounters. The discharge summary and postoperative documentation from the index surgical encounter were not available at the time of model inference and were not provided to the model. These inputs were processed by the GPT-4o–based AI engine to generate American Society of Anesthesiologists (ASA) physical status classifications and postoperative nausea and vomiting risk predictions for clinician review. PACS: picture archiving and communication system.

The AI system comprised three functional layers: (1) a hospital data input layer, (2) a GPT-4o prediction engine, and (3) an output module. As depicted in Figure 1, the system collected structured and unstructured inputs from hospital information systems, including the hospital information system, nursing information system, laboratory information system, and anesthesia information management system, as well as NHI cloud data, preoperative clinical documentation, and historical summaries from prior hospital encounters when available.

All model inputs were restricted to information available before the index surgery and before the clinician completed the preanesthetic evaluation. Historical summaries from prior hospital encounters could be included as part of the longitudinal medical history when available. However, the discharge summary and postoperative documentation from the index surgical encounter were not available at the time of inference and were not provided to the model.

To prevent circularity and label leakage in the ASA agreement analysis, the anesthesiologist’s preanesthetic evaluation note and the signed anesthesia consultation record from the index surgical encounter were excluded from the model input. Additionally, any explicit clinician-assigned ASA physical status classification contained in structured fields or unstructured clinical text was excluded or masked before inference. The clinician-assigned ASA classification used as the reference standard was retrieved separately only after AI inference had been completed and was used solely for the subsequent agreement analysis.

**Figure 2.**
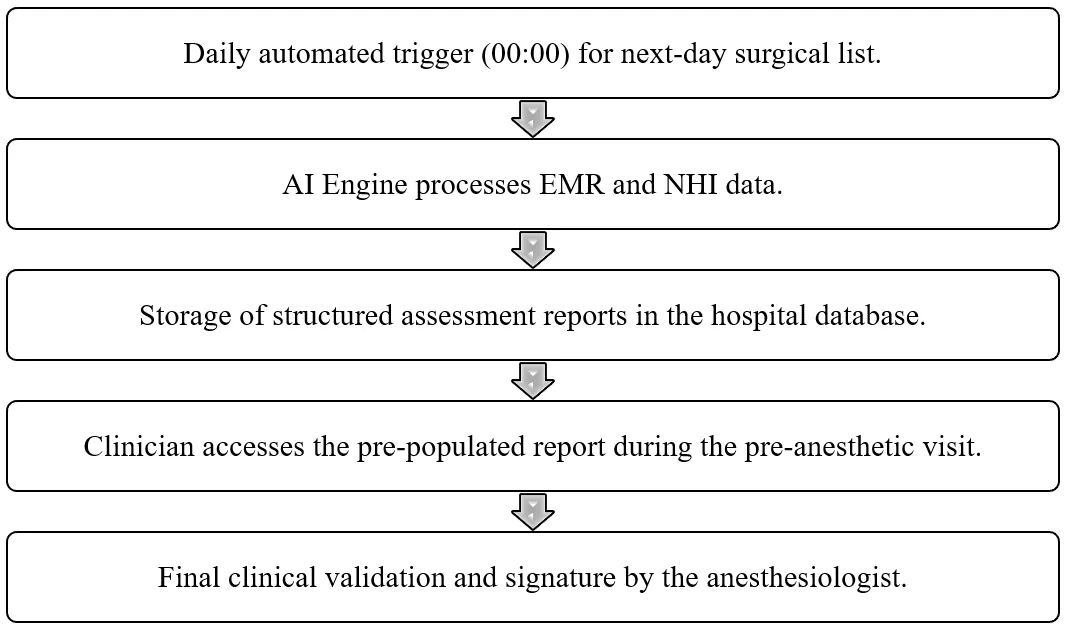
The system used an automated batch-processing and clinical retrieval workflow. The system automatically retrieves the next-day surgical schedule, processes deidentified electronic medical record (EMR) and National Health Insurance (NHI) cloud data through the GPT-4o engine, stores structured assessment reports in the hospital database, and enables anesthesiologists to review and validate the prepopulated report during the preanesthetic visit before final approval and integration into the medical record.

To optimize clinical efficiency and minimize latency, the system followed an automated batch-processing and clinical retrieval workflow (Figure 2):

1. Automated daily trigger: The system was triggered automatically at midnight each day to retrieve the surgical schedule for the following day.
2. Batch analysis: The GPT-4o engine processed the deidentified longitudinal data to generate preanesthetic assessments.
3. Secure storage: The structured reports were stored in the hospital database.
4. Clinical interaction: During the preanesthetic visit, anesthesiologists accessed the prepopulated reports as an auxiliary clinical decision-support tool.
5. Human-in-the-loop validation: All AI-generated outputs required clinical verification and a final electronic signature by the anesthesiologist before formal integration into the medical record.

### Sampling and Data Collection

During January to May 2025, a total of 11,817 surgical records were screened. After removing 100 duplicate records related to combined procedures, 11,717 unique surgical records remained. These records included 3815 emergency surgeries, 3403 outpatient or day-surgery encounters, and 4499 inpatient surgical patients. Emergency surgeries and outpatient or day-surgery encounters were excluded because the present study focused on inpatient surgical patients undergoing standard preanesthetic consultation. At our institution, cases planned for local anesthesia alone do not undergo routine preanesthetic consultation and are not included in the preanesthetic consultation workflow. Therefore, local-anesthesia-only cases were not part of the sampling frame for this validation study. In accordance with the Institutional Review Board–approved study protocol, only adult patients aged 18 years or older at the time of surgery were eligible for inclusion. Pediatric patients were not included because individuals younger than 18 years were outside the approved study population and were considered a vulnerable population. Among the 4499 inpatient surgical patients, 95 patients classified as ASA physical status V or VI were further excluded because the study system was designed to validate routine preanesthetic assessment for ASA I-IV surgical patients rather than moribund patients or organ donors. The final eligible sampling frame, therefore, consisted of 4404 inpatient surgical patients. From this sampling frame, 600 adult patients were selected using stratified simple random sampling, with 120 patients randomly selected from each month between January and May 2025. All patients included in the final analytic cohort had a completed clinician-assigned ASA classification available for comparison with the AI-generated ASA classification.

### Sample Size Justification

Because this was a retrospective validation study, the sample size was determined by feasibility and temporal balancing rather than by a formal a priori power calculation. A total of 600 patients were selected from the eligible inpatient surgical cohort, with 120 patients randomly sampled from each month between January and May 2025. This sampling strategy was used to provide balanced temporal representation and to reduce the influence of month-specific workflow or case-mix variation.

The primary validation focus was agreement between AI-generated and clinician-assigned ASA physical status classifications. The PONV analysis was treated as a secondary performance evaluation. Because PONV occurred in 49 of 600 patients, estimates of sensitivity for PONV prediction were subject to greater statistical uncertainty and were therefore interpreted cautiously.

### Prompt Development and Validation

The prompting strategy was developed through an iterative refinement process from July to December 2024. A multidisciplinary expert panel consisting of board-certified anesthesiologists and certified registered nurse anesthetists conducted multiple cycles of testing, evaluation, and refinement to optimize the model’s clinical reasoning and output structure. These iterations specifically addressed inconsistencies in extracting unstructured narratives, including smoking status and motion sickness history, and refined the logic for prioritizing objective electronic medical record (EMR) documentation over subjective patient-reported information when discrepancies were identified. The final prompt template reached expert consensus after demonstrating stable performance across diverse clinical scenarios.

For the study period, the final version was implemented with the model temperature fixed at 0 to support highly reproducible outputs. The final structured prompt template used for the GPT-4o–enabled preanesthetic assessment system is provided in Multimedia Appendix 1. Because the prompt was implemented in Traditional Chinese in the actual clinical workflow, the appendix includes the verbatim Traditional Chinese prompt and an English translation for editorial and reviewer reference. The English translation is provided for reviewer reference only and was not the version used during system execution.

### Foundation Model and Deployment Configuration

The AI engine used GPT-4o through a hospital-integrated, vendor-managed cloud deployment rather than a locally hosted model or a directly researcher-controlled API account. During the study period, the model was accessed through the same institutional deployment environment for all cases. The final prompt template, input preprocessing logic, output format requirements, and inference workflow were fixed throughout the study period and were applied identically to both the NHI-enabled and NHI-withheld conditions.

Temperature was fixed at 0 to support highly reproducible outputs. Other generation parameters, including top_p, maximum output tokens, frequency penalty, presence penalty, and system-level safety or routing parameters, were managed by the vendor deployment environment and were not directly exposed in the retrospective research dataset. No fine-tuning, local model training, or model-weight modification was performed by the study team.

Because the system was operated through a hospital-integrated deployment, the exact underlying model snapshot string and some deployment-level hyperparameters were not directly available to the investigators at the time of retrospective analysis. This limitation is reported to ensure transparency. Future prospective validation studies should retain deployment-level metadata, including model snapshot, API version, deployment version, and generation parameters, to improve reproducibility.

### Model Functionality and Outputs

The GPT-4o–enabled system processed both structured and unstructured EMR data to generate two primary outputs: (1) ASA physical status classification and (2) prediction of PONV risk (low, moderate, or high).

To simulate expert-level clinical reasoning, a structured prompt was created to guide GPT-4o’s behavior. This prompt was constructed based on a combination of institutional anesthesia practice guidelines and evidence-based criteria from the published literature.

The prompting logic was designed to:

1. Identify comorbidities based on the patient’s history, medication records, and diagnostic codes.
2. Interpret unstructured data elements, including clinical notes and surgical reports.
3. Assign ASA class II or higher if any chronic systemic disease is present.
4. Perform PONV risk stratification using an Apfel-informed prompt that instructed the model to consider 4 Apfel-related factors: female sex, nonsmoking status, history of PONV or motion sickness, and expected postoperative opioid use.
5. Generate a final model-assigned PONV risk category as low, moderate, or high risk based on available documentation and the model’s prompt-guided clinical judgment.
6. Produce a structured, tabulated output that includes the following: (1) patient demographics, (2) comorbidities with justification, (3) ASA classification with rationale, (4) the suggested anesthesia plan, (5) optional self-pay items, (6) perioperative mortality risk estimation, and (7) clinical recommendations.

To improve clinical interpretability, the structured reports were rendered through the hospital’s front-end clinical application rather than being displayed as raw model output. The GPT-4o engine generated structured text with predefined formatting markers, including Markdown bold syntax and HTML color tags for clinically significant findings. These formatting markers were parsed and rendered by the application layer to display high-risk findings in bold text or red font where supported by the interface. In plain-text or unsupported environments, the system retained bold formatting only.

### Primary Outcome and Threshold

PONV within 24 hours after surgery was classified as a binary outcome based on documentation in postoperative nursing notes, recovery records, physician progress notes, or medication records indicating nausea, vomiting, or the administration of rescue antiemetic therapy. Because the outcome was retrospectively ascertained from routine clinical documentation, undocumented mild or transient PONV episodes may not have been captured.

### Evaluation of AI Performance

The performance of the AI system was evaluated by comparing ASA physical status classifications from three sources: (1) clinician-assigned ASA classification, (2) AI-generated ASA classification without NHI data, and (3) AI-generated ASA classification with NHI data. The clinician-assigned ASA classification was used as the clinical reference classification for agreement analysis.

To evaluate the incremental contribution of NHI cloud data, each of the same 600 patients was processed under 2 input conditions. In the NHI-enabled condition, the model input included institutional EMR data and the NHI cloud data block. In the NHI-withheld condition, the NHI cloud data block was completely removed before model inference, while all other institutional EMR inputs, prompt instructions, model settings, and output requirements were kept identical. Therefore, the comparison between AI-generated ASA classifications with and without NHI data was based on paired outputs from the same patients rather than on different patient cohorts.

Agreement between AI-generated and clinician-assigned ASA classifications was assessed using Cohen κ coefficient with 95% CIs. Differences in model performance with and without NHI data were summarized descriptively.

For PONV risk prediction, the AI system generated a final risk category of low, moderate, or high risk. The PONV component was implemented as an Apfel-informed, prompt-based clinical risk stratification task rather than as a deterministic recalculation of the conventional Apfel score by the investigators. The prompt instructed the model to consider 4 Apfel-related factors: female sex, nonsmoking status, history of PONV or motion sickness, and expected postoperative opioid use. However, the risk category analyzed in this study was extracted directly from the model-generated output rather than recalculated after inference by rule-based criterion counting.

These predicted risk levels were compared with actual postoperative PONV events documented in clinical records within 24 hours after surgery. Diagnostic performance was evaluated using sensitivity, specificity, overall accuracy, positive predictive value, negative predictive value, and confusion matrix analysis. The relationship between the model-assigned PONV risk group and observed PONV outcome was analyzed using the 2-sided Fisher-Freeman-Halton exact test because the expected cell-count assumption for the Pearson chi-square test was not satisfied. The strength of the association was summarized using Cramér V.

Because the GPT-4o system generated ordinal PONV risk categories rather than calibrated continuous probability scores, receiver operating characteristic (ROC) and precision-recall plots were constructed using the available categorical thresholds. The primary threshold defined high risk as test-positive, whereas the sensitivity analysis defined moderate and high risk as test-positive. These analyses were interpreted as categorical threshold–based performance summaries rather than a formal optimization of a continuous probability threshold. The supplementary ROC and precision-recall plots are provided in Multimedia Appendix 2.

### Statistical Analysis

Data management, data checking, variable coding, descriptive summaries, and tabulation were performed using Microsoft Excel 365. Inferential statistical analyses were conducted using SPSS Statistics (version 29.0.1.1; IBM Corp). Agreement between AI-generated and clinician-assigned ASA classifications was assessed using Cohen κ coefficient with 95% CIs. The association between model-assigned PONV risk categories and observed PONV outcomes was evaluated using the 2-sided Fisher-Freeman-Halton exact test because more than 20% of the contingency-table cells had expected counts below 5. The strength of the association was summarized using Cramér V. Diagnostic performance for PONV prediction was summarized using sensitivity, specificity, overall accuracy, positive predictive value, negative predictive value, and confusion matrix analysis. All hypothesis tests were 2-tailed, and a *P*<.05 was considered statistically significant. Descriptive statistics were used to summarize categorical variables and classification outputs. No imputation was performed for missing outcome or classification data.

### Structured Prompt Logic for GPT-4o–Enabled Preanesthetic Assessment

#### Prompt Design Objectives

To emulate the clinical reasoning of an experienced anesthesiologist, we developed a structured prompt to guide the behavior of the GPT-4o LLM. The prompt was constructed using institution-specific clinical heuristics, guideline-derived decision rules, and explicit formatting instructions. Its purpose was to enable the model to generate a complete, clinically relevant preanesthetic assessment based on both structured and unstructured data retrieved from the EMR.

The prompt was designed to perform the following functions: (1) evaluate comorbidities and clinical history based on EMR data, (2) derive the ASA physical status classification in accordance with ASA guidelines, (3) stratify PONV risk using an Apfel-informed, prompt-based approach, (4) recommend appropriate anesthesia modalities and procedural interventions (eg, an arterial line and a central venous catheter placement), (5) assess the feasibility of optional self-pay anesthesia services, (6) estimate the perioperative mortality risk, and (7) suggest referrals for additional preoperative consultations when clinically indicated.

#### Clinical Reasoning Logic

##### Comorbidity Recognition

When discrepancies were found between patient self-reports and EMR data (such as *International Classification of Diseases, 10th Revision* [*ICD-10*] codes, medication history, or laboratory abnormalities), the model prioritized objective documentation. If any chronic systemic disease was identified, the system defaulted to assigning an ASA classification of at least class II, consistent with ASA definitions.

##### PONV Risk Stratification

The PONV component was designed as an Apfel-informed prompt-based risk stratification task. The prompt instructed the model to consider 4 Apfel-related factors associated with PONV: female sex, nonsmoking status, history of motion sickness or previous PONV, and expected postoperative opioid use. Additionally, the prompt asked the model to generate a final low-risk, moderate-risk, or high-risk classification according to the expected clinical incidence and to provide reasons for high-risk factors.

Because the implemented prompt contained both Apfel-related factor instructions and an incidence-based risk classification instruction, the final PONV risk category was treated as a model-assigned ordinal risk category rather than as a deterministic recalculation of the conventional Apfel score. Accordingly, the low, moderate, and high categories in this study should be interpreted as prompt-defined clinical decision-support categories rather than as standard Apfel score categories.

### Clinical Output Structure

The model was configured to return structured, tabulated summaries across the following clinical domains:

1. Demographics: Includes blood type, sex, age, and year of birth.
2. Comorbidities and surgical history: Includes conditions such as diabetes, cardiovascular disease, thromboembolism, respiratory illnesses, hepatic or renal dysfunction, prior surgeries, substance use, allergies, psychiatric disorders, and a family history of malignant hyperthermia.
3. ASA classification: Displays the assigned ASA physical status along with the supporting rationale.
4. Anesthesia plan: Recommends an anesthesia modality (eg, endotracheal tube general anesthesia, intravenous general anesthesia, spinal anesthesia, epidural anesthesia, and local anesthesia) and flags indications for invasive monitoring (eg, arterial line and central venous catheter), intensive care unit admission, or fiberoptic airway evaluation.
5. Optional patient-pay services: Suggests nonreimbursed perioperative options (eg, BIS monitor, patient-controlled analgesia, and warming devices), based on surgical type, patient age, and comorbidity profile.
6. Perioperative mortality estimation: Provides a risk estimate based on cumulative clinical factors.
7. Referral suggestions: Identifies cases requiring additional evaluation or a specialty consultation (eg, cardiology).

### Formatting Protocol

To enhance interpretability, clinically significant findings (eg, positive comorbidities or high-risk status) are rendered in bold text. In environments that support HTML or rich-text formatting, such findings are also highlighted in red font using a <span style=“color:red”>text</span >tag. In plain-text contexts (eg, terminal or PDF output), only bold formatting is applied.

## Results

### Baseline Characteristics and Binary-Coded Apfel-Related Factors

A total of 600 adult surgical patients were included in the analysis. The results are presented in two parts: (1) the agreement between clinician-assigned and AI-generated ASA physical status classifications and (2) the performance of AI-based risk stratification for PONV.

Baseline characteristics and binary-coded Apfel-related factors are shown in Table 1. The cohort included adults aged 18 to 80 years. Apfel-related factors were coded as positive when identified from the model input or the preoperative clinical record review and as not positive when they were not identified in the available preoperative records. Among the 600 patients, 161 (26.8%) were female, 26 (4.3%) had nonsmoking status documented, 20 (3.3%) had a documented history of previous PONV or motion sickness, and 20 (3.3%) had expected postoperative opioid use documented. The relatively low coded frequencies of nonsmoking status, previous PONV or motion sickness, and expected postoperative opioid use likely reflect incomplete or nonexplicit documentation in the available preoperative records rather than their true prevalence in the study cohort.

**Table 1.** Baseline characteristics and binary-coded Apfel-related factors of the study cohort^a^.

Characteristic	Overall cohort (N=600)
Age (y), mean (SD)	42.8 (16.2)
Age (y), median (IQR)	41.0 (30.0‐53.0)
Age range (y)	18‐80
Age group (y), n (%)	
<18	0 (0)
18‐64	526 (87.7)
≥65	74 (12.3)
Sex, n (%)	
Male	439 (73.2)
Female	161 (26.8)
Binary-coded Apfel-related positive factors, n (%)	
Female sex	161 (26.8)
Nonsmoking status	26 (4.3)
History of PONV^b^ or motion sickness	20 (3.3)
Expected postoperative opioid use	20 (3.3)
Number of binary-coded Apfel-related positive factors, n (%)	
0	422 (70.3)
1	151 (25.2)
2	6 (1)
3	20 (3.3)
4	1 (0.2)

a Apfel-related factors were coded as binary variables based on information available in the model input and preoperative clinical record review. A value of 1 indicated that the factor was identified as positive, whereas a value of 0 indicated that the factor was not identified as positive in the available preoperative records. The number of binary-coded Apfel-related positive factors is reported descriptively and was not used by the investigators to recalculate the AI-generated PONV risk category after inference.

b PONV: postoperative nausea and vomiting.

### ASA Classification Agreement

Table 2 summarizes the distribution of ASA classifications across 3 sources: clinician-assigned ASA classifications, AI-generated classifications with NHI data, and AI-generated classifications without NHI data. Among the 600 patients, the majority of clinician-assigned ASA classifications were ASA II (n=498 patients, 83%), followed by ASA III (n=57 patients, 9.5%), ASA I (n=39 patients, 6.5%), and ASA IV (n=6 patients, 1%).

**Table 2.** Distribution of American Society of Anesthesiologists (ASA) classifications by clinicians and AI models with and without National Health Insurance (NHI) data.

ASA grade	Clinician-assigned ASA	AI with NHI	AI without NHI
1	39	37	140
2	498	497	409
3	57	62	48
4	6	4	3
Total patient number	600	600	600

The AI system incorporating NHI data closely matched clinicians’ assessments, assigning ASA I to 37 patients and ASA II to 497 patients. In contrast, the AI system without NHI data substantially overestimated ASA I classifications (140 vs 39 patients; +101) and underestimated ASA II classifications (409 vs 498 patients; –89), indicating a systematic underestimation of preoperative physical status severity when NHI cloud data were unavailable.

As shown in Table 3, agreement between AI-generated and clinician-assigned ASA classifications was high when NHI data were incorporated, with a Cohen κ of 0.883 (95% CI 0.841‐0.946). In contrast, agreement was lower when NHI data were not incorporated, with a Cohen κ of 0.518 (95% CI 0.412‐0.691), indicating moderate agreement. The difference in κ values (Δκ=0.365) suggests that incorporating NHI cloud data improved consistency between AI-generated and clinician-assigned ASA classifications.

**Table 3.** Agreement between AI-generated and clinician-assigned ASA^a^ classifications.

Comparison	Cohen κ	95% CI^b^
AI with NHI^c^ vs clinician-assigned ASA	0.883	0.841‐0.946
AI without NHI vs clinician-assigned ASA	0.518	0.412‐0.691
Difference in κ	0.365	—^d^

a ASA: American Society of Anesthesiologists.

b The 95% CI was calculated for each Cohen κ estimate. A 95% CI for the difference in κ was not calculated.

c NHI: National Health Insurance.

d Not applicable.

### PONV Risk Prediction Performance

The performance of the AI system in predicting PONV is shown in Table 4. A total of 8.2% (49/600) patients experienced PONV. The AI model stratified patients into 3 risk levels: low, moderate, and high. These risk groups represented the final AI-generated, Apfel-informed, prompt-based classifications and were not recalculated by the investigators using deterministic Apfel criterion counting after model inference.

**Table 4.** PONV^a^ prediction vs actual outcomes (primary threshold: high risk=test-positive)^b^.

PONV outcome	Low risk	Moderate risk	High risk	Total
No PONV	540	6	5	551
PONV	27	5	17	49
Total	567	11	22	600

a PONV: postoperative nausea and vomiting.

b The association between the 3 model-assigned PONV risk categories and observed PONV outcomes was evaluated using the 2-sided Fisher-Freeman-Halton exact test (*P*<.001) because more than 20% of the contingency-table cells had expected counts below 5. Cramér V was used to summarize the strength of the association (V=0.531). The primary diagnostic-performance analysis counted high risk as test-positive, whereas the sensitivity analysis counted moderate and high risk as test-positive. In the sensitivity analysis, sensitivity was 44.9%, specificity was 98.0%, positive predictive value was 66.7%, and negative predictive value was 95.2%.

Among the 49 patients who developed PONV, the model classified 17 as high risk, 5 as moderate risk, and 27 as low risk. Among the 551 patients who did not develop PONV, the model classified 540 as low risk, 6 as moderate risk, and 5 as high risk.

Using high-risk as the primary test-positive threshold, the model achieved a sensitivity of 34.7% (95% CI 22.9‐48.7), specificity of 99.1% (95% CI 97.9‐99.6), positive predictive value of 77.3%, and negative predictive value of 94.5%. The observed incidence of PONV differed significantly across the 3 model-assigned risk categories (2-sided Fisher-Freeman-Halton exact test, *P*<.001), with a large association as indicated by Cramér V of 0.531. The incidence of PONV was 4.8% (27/567) in the low-risk group, 45.5% (5/11) in the moderate-risk group, and 77.3% (17/22) in the high-risk group.

In the sensitivity analysis counting the moderate-risk and high-risk categories as test-positive, sensitivity increased to 44.9% (95% CI 31.9‐58.7), while specificity was 98.0% (95% CI 96.5‐98.9), positive predictive value was 66.7%, and negative predictive value was 95.2%. Supplementary ROC and precision-recall plots based on ordinal PONV risk thresholds are provided in Multimedia Appendix 2. Because the model generated categorical low-risk, moderate-risk, and high-risk levels rather than calibrated continuous probabilities, these plots were constructed using the available categorical thresholds.

## Discussion

### Principal Findings

This retrospective validation study showed that a GPT-4o–enabled preanesthetic assessment system can generate clinically meaningful ASA physical status classifications and PONV risk predictions using both structured and unstructured clinical data. The primary validation focus was agreement between AI-generated and clinician-assigned ASA physical status classifications. The most important finding was that agreement between AI-generated ASA classifications and anesthesiologist-assigned classifications was high when NHI cloud data were incorporated (κ=0.883, 95% CI 0.841‐0.946), whereas agreement was lower without NHI data (κ=0.518, 95% CI 0.412‐0.691). The observed reduction in agreement (Δκ=0.365) highlights the importance of comprehensive and longitudinal patient information in AI-assisted perioperative risk assessment.

For PONV prediction, the model showed a different performance profile. Using the high-risk category as the primary positive threshold, specificity was very high (99.1%), whereas sensitivity was modest (34.7%). The primary threshold also yielded a positive predictive value of 77.3% and a negative predictive value of 94.5%. When the test-positive threshold was broadened to include both moderate and high-risk categories, sensitivity increased to 44.9%, but the positive predictive value decreased to 66.7%, illustrating the tradeoff between identifying more potential PONV cases and preserving prediction precision. Because the system produced categorical low, moderate, and high PONV risk outputs rather than calibrated probability scores, the ROC and precision-recall plots should be interpreted as ordinal threshold–based performance summaries rather than as continuous probability–based threshold optimization. Accordingly, the PONV component should be interpreted as an ordinal risk stratification tool that identified a small high-risk subgroup with a high observed PONV incidence, rather than as a stand-alone screening system for all patients who may develop PONV.

Taken together, these findings suggest that LLM-based systems can approximate local clinicians’ reasoning in preanesthetic evaluation, particularly for ASA classification, but their performance is strongly influenced by the completeness of the underlying data environment.

### Comparison With Prior Work

The high concordance observed in ASA classification is consistent with prior work showing that AI and machine learning approaches can support or approximate ASA assignment using EHR-derived data. Previous studies have reported moderate agreement between a machine learning–derived ASA score and anesthesiologist ratings, as well as strong agreement between ChatGPT-4 (OpenAI) and expert anesthesiologists in a prospective multicenter evaluation [1,2].

Human-to-human agreement provides an important benchmark for interpreting AI-clinician agreement in ASA physical status classification. Prior studies have reported only fair-to-moderate agreement among clinicians. Riley et al [16] reported a κ value of 0.40 among 151 anesthetists assigning ASA classifications to standardized hypothetical adult patient histories. In a large clinical-practice cohort, Sankar et al [17] reported a weighted κ of 0.61 when comparing ASA physical status ratings assigned in the preoperative assessment clinic and operating theater, with 67.0% (7279/10864) of patients assigned the same ASA physical status class. Kwa et al [18] reported moderate concordance between surgeon-assigned and anesthesiologist-assigned ASA classifications in 46,284 elective surgical patients, with a weighted Cohen κ of 0.53. In this context, the agreement observed between the GPT-4o system using NHI data and anesthesiologist-assigned ASA classifications in the present study (κ=0.883) appears high relative to previously reported human-to-human agreement benchmarks. However, this comparison should be interpreted cautiously because the prior studies differed in rater design, case mix, clinical setting, and the use of weighted vs unweighted κ. Therefore, our finding should not be interpreted as evidence that AI outperforms clinicians, but rather as suggesting that comprehensive longitudinal data integration may support more consistent and standardized ASA classification.

Our findings extend this prior work in 2 ways. First, the present system was designed to function within a real clinical workflow using both structured and narrative data, rather than relying solely on predefined structured inputs. Second, the marked improvement observed after incorporation of NHI cloud data suggests that data integration, rather than model architecture alone, may be a major determinant of clinical utility.

This interpretation is further supported by the observed distributional shift in ASA predictions without NHI data. In the absence of cloud-based information, the system overassigned ASA I and underassigned ASA II, indicating a tendency to classify patients as healthier when longitudinal comorbidity data were incomplete. From a clinical perspective, this pattern is important because underestimation of ASA status could lead to insufficient preoperative preparation or underrecognition of perioperative risk. The benefit of richer preoperative data is also consistent with prior perioperative AI studies showing that the combination of structured variables and unstructured clinical text can improve prediction performance, particularly for outcomes influenced by complex comorbidity patterns [5].

Several factors may explain the limited sensitivity for PONV prediction. The prompting framework was informed by Apfel-related factors, including female sex, nonsmoking status, history of motion sickness or previous PONV, and postoperative opioid use [4]. However, the final PONV risk category evaluated in this study was the model-assigned ordinal output rather than a deterministic recalculation of the conventional Apfel score. Some of these variables are not consistently or reliably documented in routine EHRs. In particular, motion sickness history, prior PONV, and smoking status may be incompletely captured, leading to systematic underidentification of risk even when the prompt logic is otherwise clinically reasonable.

These findings are consistent with more recent work suggesting that machine learning approaches may outperform classical PONV scores when broader perioperative predictors are available [11]. Accordingly, future improvement in LLM-based PONV prediction may require not only prompt refinement but also broader feature integration, including anesthetic technique, intraoperative medication exposure, recovery-phase variables, and a more complete capture of patient-reported risk factors.

### Ethical and Regulatory Considerations

An important feature of this system is that it was developed as a workflow-oriented clinical support tool rather than as an autonomous decision-making system. The model processed deidentified data in batch mode before the preanesthetic visit, generated structured outputs for clinician review, and required final verification and an electronic signature by an anesthesiologist before their formal integration into the medical record. This human-in-the-loop design is essential because clinician acceptance of AI-generated content does not necessarily guarantee correctness [13]. More broadly, the literature on human-AI collaboration suggests that AI is most appropriate when used to augment expert interpretation rather than replace professional judgment [12].

The system also generated additional clinical outputs, including suggested anesthesia plans, indications for invasive monitoring, perioperative mortality estimates, referral suggestions, and optional patient-pay anesthesia services. These outputs were intended to support structured clinical review but were not independently validated in the present study. This distinction is important because recommendations concerning invasive monitoring, referrals, mortality risk, or nonreimbursed services may have clinical, ethical, and financial implications. These outputs should therefore be interpreted as preliminary decision-support suggestions requiring clinician verification rather than as validated automated recommendations.

The inclusion of optional patient-pay anesthesia services requires particular ethical caution. In the present system, AI-generated patient-pay items were not autonomous recommendations, were not automatically prescribed, and were not directly presented to patients without clinician review. The system identified potentially applicable services based on predefined clinical factors, including anesthesia type, surgical procedure, age, and comorbidity profile. Final assessment of clinical appropriateness, discussion with the patient, and any subsequent recommendation remained the responsibility of the anesthesiologist.

The financial-hardship logic functioned as a clinician-facing caution rather than as an automatic exclusion rule. When the medical record contained unpaid medical bills, social work documentation, physician orders, or other information indicating financial difficulty, the system alerted the reviewing clinician to consider the potential financial burden associated with nonreimbursed services. This alert did not remove patient-pay options from the clinical workflow, prohibit clinicians from discussing them, or prevent patients from considering potentially beneficial services.

The intent of this logic was partly protective, aiming to reduce indiscriminate promotion of costly nonreimbursed services to patients experiencing financial difficulty. However, we acknowledge that even a clinician-facing socioeconomic alert could influence whether a potentially beneficial option is discussed and could therefore raise concerns regarding equity and patient autonomy. The decision to introduce a patient-pay option remained based on its clinical relevance and was made by the responsible anesthesiologist after consideration of the patient’s clinical condition, preferences, and individual circumstances. When an option was considered clinically appropriate, the clinician could still provide balanced information regarding its rationale, expected benefits, alternatives, risks, and costs, thereby allowing the patient to participate in shared decision-making.

Optional patient-pay suggestions should therefore be treated as preliminary clinical prompts rather than as revenue-oriented recommendations or automatic eligibility determinations. To reduce the risk of systematic disadvantage, appropriate governance should include institutionally approved criteria, multidisciplinary oversight, clinician education, and periodic audits to assess whether patients with financial-risk alerts are systematically less likely to receive information about clinically relevant options. Review of clinician responses to these alerts and documentation of subsequent discussions may further support transparency and accountability. These safeguards are important for preserving equity, patient autonomy, and shared decision-making.

More broadly, to reduce the risk of automation bias, particularly among junior clinicians, AI-supported workflows should be accompanied by appropriate training in AI literacy, information security, and critical interpretation of model outputs. Clinical accountability for final assessment and decision-making should remain with the responsible anesthesiologist.

### Limitations

First, this was a single-center retrospective study, and model performance may differ in other hospitals, documentation systems, or patient populations. The present system benefited from the NHI cloud infrastructure in Taiwan, which provides longitudinal claims and medication information beyond a single-institution record. Although the underlying principle of longitudinal data integration is transferable, comparable performance may not be achieved in settings with less complete data interoperability. Future multicenter validation is needed across institutions with different documentation cultures, coding practices, and data completeness.

Second, the comparison between NHI-enabled and NHI-withheld inputs revealed a clinically important default-to-healthy bias when longitudinal NHI data were unavailable. Specifically, when NHI data were withheld, the system classified 140 patients as ASA I, whereas clinicians classified only 39 patients as ASA I. This pattern suggests that, in the absence of longitudinal comorbidity and medication information, the model may underrecognize systemic disease burden and classify patients as healthier than they are. From a patient safety perspective, such underclassification could lead to underrecognition of perioperative risk if clinicians accept AI-generated outputs without careful review. This finding reinforces the need for mandatory anesthesiologist verification, clear labeling of data completeness, and caution when using AI-generated ASA classifications in settings where longitudinal external health data are unavailable.

Third, the reference standard for ASA classification was clinician-assigned ASA status rather than an adjudicated consensus panel. Therefore, the observed agreement reflects concordance with local real-world clinical practice rather than agreement with an objective gold standard. In routine clinical workflow, independent duplicate ASA assessments by multiple anesthesiologists are not systematically performed for the same patient; therefore, an internal human-to-human κ could not be calculated from this retrospective dataset. Because ASA classification is known to vary across providers, the model should be interpreted as approximating local clinician judgment rather than establishing an independent ground truth.

Fourth, the structured prompt included a conservative rule assigning ASA class II or higher when a chronic systemic disease was identified. This rule was intended to reduce the risk of underrecognizing clinically relevant comorbidity during automated preanesthetic assessment. However, it may not fully capture cases in which a prior localized condition has completely resolved or has no current systemic implications. In such cases, an ASA I classification may still be clinically appropriate. This hardcoded logic therefore represents an algorithmic constraint and may contribute to conservative upward classification at the ASA I-II boundary, particularly when historical diagnoses are not clearly distinguishable from active systemic disease in the source clinical records. Future versions of the prompt should more explicitly distinguish active systemic disease from remote, resolved, or clinically insignificant medical history.

Fifth, the PONV component should be interpreted with caution because it was implemented as an Apfel-informed, prompt-based clinical risk stratification task rather than as a deterministic recalculation of the conventional Apfel score. The prompt instructed the model to consider 4 Apfel-related factors: female sex, nonsmoking status, history of PONV or motion sickness, and expected postoperative opioid use. However, the analyzed PONV risk category was extracted from the model’s final low-risk, moderate-risk, or high-risk output rather than recalculated by the investigators using rule-based Apfel criterion counting after inference. Therefore, the PONV results represent the performance of the study system’s prompt-defined ordinal risk stratification rather than a validation of standard Apfel score thresholds.

The prompt also contained both Apfel-related factor instructions and an incidence-based risk classification instruction. This prompt-design ambiguity may have contributed to the high proportion of low-risk classifications and should be addressed in future prompt refinement and prospective validation. Additionally, some Apfel-related variables, including smoking status, prior PONV, motion sickness history, and expected postoperative opioid use, may not have been consistently documented in routine EHRs, which may have further limited sensitivity.

PONV events were retrospectively identified from routine clinical records. Mild or transient nausea and vomiting may not have been consistently documented, meaning that the true incidence of PONV may have been higher than the observed 8.2% (49/600). This ascertainment bias may have influenced estimates of diagnostic performance and may have caused some apparent AI false positives to represent undocumented true-positive events.

Sixth, the sample size was determined by feasibility and temporal balancing rather than by a formal a priori power calculation. Although the 600-patient cohort allowed the evaluation of ASA agreement in a temporally balanced retrospective sample, the study was not specifically powered for PONV prediction. Only 49/600 (8.2%) patients experienced documented PONV within 24 hours after surgery. As a result, sensitivity estimates for PONV prediction had wider uncertainty and should be interpreted cautiously. Future studies with larger samples or enriched PONV-positive cohorts are needed to more precisely evaluate model performance for PONV risk prediction.

Seventh, while the temperature parameter was fixed at 0 to improve output consistency, the system was operated through a hospital-integrated, vendor-managed cloud deployment. Therefore, the exact underlying model snapshot string and some deployment-level hyperparameters were not directly available in the retrospective research dataset. LLM outputs may also be influenced by prompt framing, source documentation quality, model updates, deployment configuration, and computational variation. This setting should therefore be described as supporting highly reproducible outputs rather than guaranteeing strict determinism. Future prospective studies should retain deployment-level metadata, including model snapshot, API version, deployment version, and generation parameters, to improve reproducibility.

Eighth, the study evaluated only ASA classification agreement and PONV risk stratification. Other generated outputs, including suggested anesthesia plans, invasive monitoring recommendations, perioperative mortality estimation, referral suggestions, and optional patient-pay anesthesia services, were not independently validated. These outputs should therefore be interpreted as preliminary decision-support prompts requiring anesthesiologist verification rather than validated automated clinical recommendations.

Ninth, although the system was designed to support workflow efficiency through automated batch processing and prepopulated reporting, operational outcomes such as documentation time, assessment completion time, clinician workload, user satisfaction, and downstream decision-making were not directly measured. Therefore, the present findings support diagnostic and workflow feasibility but do not yet demonstrate measurable efficiency gains or improved clinical outcomes.

### Future Directions

Future studies should validate this system prospectively and across multiple clinical sites. Particular attention should be paid to improving the sensitivity of PONV prediction by incorporating additional perioperative predictors, including anesthetic technique, intraoperative medication exposure, postoperative opioid use, recovery-room symptoms, and structured patient-reported histories of motion sickness or previous PONV. Future work should also evaluate representative false-negative and false-positive cases in greater detail to identify whether errors are driven primarily by model reasoning, incomplete documentation, or outcome underascertainment.

Additional validation is also needed for clinical outputs beyond ASA and PONV, including suggested anesthesia plans, indications for invasive monitoring, perioperative mortality estimation, referral suggestions, and optional patient-pay service recommendations. Future implementation studies should measure effects on documentation time, clinician workload, assessment consistency, patient safety, prophylaxis decisions, and cost-related ethical outcomes.

### Conclusions

This study demonstrated the feasibility of a GPT-4o–powered AI system for preanesthetic assessment using structured and unstructured data from EHRs and NHI cloud records. The system showed high agreement with anesthesiologist-assigned ASA classifications when NHI data were incorporated, supporting the value of comprehensive longitudinal data for AI-assisted surgical risk stratification. For PONV prediction, the model demonstrated high specificity and high negative predictive value but modest sensitivity, indicating that it identified a small high-risk subgroup with a high observed incidence of PONV but did not capture all patients who developed PONV.

These findings support the use of LLM-based clinical decision-support tools as adjuncts to, rather than replacements for, anesthesiologist judgment. The system may help synthesize heterogeneous clinical information, standardize preliminary assessment, and surface clinically relevant findings for clinician confirmation. Further prospective and multicenter studies are needed to improve PONV sensitivity, validate additional clinical outputs, evaluate ethical safeguards for optional patient-pay recommendations, and quantify real-world effects on workflow efficiency and clinical decision-making.

## Supplementary material

10.2196/83893 Multimedia Appendix 1 Verbatim structured prompt template used for the GPT-4o–enabled preanesthetic assessment system.

10.2196/83893 Multimedia Appendix 2 Ordinal threshold–based receiver operating characteristic and precision-recall plots for postoperative nausea and vomiting prediction.

## Abbreviations

ASA: American Society of Anesthesiologists
EHR: electronic health record
EMR: electronic medical record
*ICD-10*: *International Classification of Diseases, 10th Revision*
ICU: intensive care unit
LLM: large language model
NHI: National Health Insurance
PONV: postoperative nausea and vomiting
ROC: receiver operating characteristic
TRIPOD: Transparent Reporting of a multivariable prediction model for Individual Prognosis or Diagnosis
