# Supplementary material for: GPT-4o–Powered Preanesthetic AI: Development and Validation Study
Source: JMIR Med Inform. 2026 Aug 25;14:e83893. doi: 10.2196/83893 (PMC13506160; doi:10.2196/83893)
Supplement: Supplementary file 2 [file medinform-v14-e83893-s002.docx]

Multimedia Appendix 2

Receiver Operating Characteristic and Precision-Recall Plots for Postoperative Nausea and Vomiting Prediction Based on Ordinal Risk Thresholds

This appendix provides supplementary receiver operating characteristic (ROC) and precision-recall plots for postoperative nausea and vomiting (PONV) prediction using the GPT-4o–enabled pre-anesthetic assessment system.

The GPT-4o system generated ordinal PONV risk categories rather than calibrated continuous probability scores. Specifically, patients were classified into Low, Moderate, or High risk groups. Therefore, the ROC and precision-recall plots were constructed using the available categorical thresholds instead of continuous probability cutoffs. The primary threshold defined High risk as test-positive, whereas the sensitivity analysis defined Moderate and High risk as test-positive.

**Table S1. PONV prediction versus actual outcomes.**

| **PONV Outcome** | **Low risk** | **Moderate risk** | **High risk** | **Total** |
| --- | --- | --- | --- | --- |
| No PONV | 540 | 6 | 5 | 551 |
| PONV | 27 | 5 | 17 | 49 |
| Total | 567 | 11 | 22 | 600 |

For the primary threshold, in which High risk was considered test-positive, the model identified 17 true-positive cases and 5 false-positive cases, with sensitivity of 34.7%, specificity of 99.1%, positive predictive value of 77.3%, and negative predictive value of 94.5%.

For the secondary threshold, in which Moderate and High risk were considered test-positive, the model identified 22 true-positive cases and 11 false-positive cases, with sensitivity of 44.9%, specificity of 98.0%, positive predictive value of 66.7%, and negative predictive value of 95.2%.

ROC plot based on ordinal PONV risk thresholds

Sensitivity

| False positive rate (1 − specificity) | Sensitivity |
| --- | --- |
| 0 | 0 |
| 0.009 | 0.347 |
| 0.02 | 0.449 |
| 1 | 1 |

| False positive rate (1 − specificity) | Random classifier |
| --- | --- |
| 0 | 0 |
| 1 | 1 |

**Figure S1A. ROC plot based on ordinal PONV risk thresholds.**

Precision-recall plot based on ordinal PONV risk thresholds

Precision (positive predictive value)

| Recall (sensitivity) | Precision |
| --- | --- |
| 0.347 | 0.773 |
| 0.449 | 0.667 |
| 1 | 0.082 |

| Recall (sensitivity) | Observed PONV prevalence |
| --- | --- |
| 0 | 0.082 |
| 1 | 0.082 |

**Figure S1B. Precision-recall plot based on ordinal PONV risk thresholds.**

Figure S1. Receiver operating characteristic and precision-recall plots for postoperative nausea and vomiting prediction based on ordinal Low, Moderate, and High risk categories. Because the GPT-4o system generated categorical risk outputs rather than calibrated continuous probability scores, the plots were constructed using available categorical thresholds. The diagonal dotted line in the ROC plot represents random-classifier performance, and the horizontal dotted line in the precision-recall plot represents the observed PONV prevalence of 8.2%.

Because the system did not output calibrated continuous probabilities, these plots should be interpreted as categorical threshold–based performance summaries rather than formal optimization of a continuous probability threshold. The plots illustrate the tradeoff between the two clinically interpretable thresholds: lowering the test-positive threshold from High risk alone to Moderate or High risk increased sensitivity from 34.7% to 44.9%, but reduced positive predictive value from 77.3% to 66.7%.
